# Coding and Validation for Breadth and Desirability of 1,214 English Adjectives

**DOI:** 10.1038/s41597-026-06934-9

**Published:** 2026-03-03

**Authors:** Lin L. Lin, Rick Dale, Steven J. Stroessner

**Affiliations:** Department of Communication, University of California, Los Angeles, USA

**Keywords:** Communication, Psychology, Interdisciplinary studies

## Abstract

Adjectives are essential in how people describe, evaluate, and reason about others. They differ along meaningful semantic dimensions such as desirability (e.g., “friendly” is more positive than “rude”) and breadth (e.g., “punctual” is narrower than “reliable”). Adjectival breadth has received limited empirical attention, partly because existing resources are sparse and outdated. We introduce a new database with subjective ratings from approximately 1,500 Americans for 1,214 adjectives on both breadth and desirability. Unlike existing resources, this updated database is more comprehensive and diverse, allowing for detailed analysis of adjectival use in academic and applied contexts. We validate this database with a large-scale analysis of online product reviews, showing how variation in adjective breadth is a common feature of natural language use. This database should prove valuable for research on semantic representation, social inference, and evaluative communication across various fields.

## Background & Summary

Language plays a crucial role in many cognitive and social processes of humans, including how they think, reason, communicate, and interact. It forms the basis for inference because it not only enables communication but also offers a framework for understanding meaning and psychological states^1,2^. Additionally, it acts as a social marker for affiliation and social interaction^3–8^ and serves as a channel for social and cultural learning^9,10^. Because of its importance in fundamental mental and social functions, language is a central focus in fields such as anthropology, communication, psychology, sociology, and marketing.

People often exhibit subtle linguistic biases in their speech, frequently without conscious awareness or intent. The linguistic category model^11^ (LCM) was developed to account for how variations in linguistic descriptions can lead to different interpretations of events and people. The LCM argues that the same event or behavior can be expressed with different words, each possibly conveying different social messages. For example, observing someone kick another person can be described using specific action verbs (e.g., “kick”), more interpretive verbs (e.g., “attack”), or very general state verbs (e.g., “hate”). Research using the LCM shows that levels of abstraction in descriptions allow message receivers to draw different conclusions about a person^12–15^. As the level of abstraction increases, it appears to provide more information about the individual, which can lead to stronger expectations about their behavior across time and situations. However, more abstract descriptions make it harder to verify if statements are true and to imagine counterexamples.

Finding cues in linguistic patterns has long been a focus in applied natural language processing, extending from psycholinguistics to education^16,17^. Over decades, studies on the LCM have shown interesting variations in language patterns, especially across different social contexts. However, this research mainly concentrates on differences in verb abstractness, with only two studies^18,19^ considering how adjective breadth varies. All languages contain adjectival word classes^20^, which play a key role in attribution in English and related languages, especially in psychology and psycholinguistics. Adjectives are a primary way to communicate detail (e.g., “big,” “old,” “red”)^21^ and form the semantic basis of stereotypes when describing social groups (e.g., “lazy,” “rude,” “aggressive”)^22^. According to the LCM, adjectives are considered the most abstract category, but there are significant differences in their breadth^23^. These differences could be very important because they may greatly affect both message senders and receivers. For example, a lab member who arrives on time could be described as either “punctual” or “reliable.” The former is narrow and specific to one act, while the latter is broad and implies much more about the person across different contexts and over time. Additionally, varying levels of adjectival breadth offer an indirect way to measure self-esteem using the Breadth–based Adjective Rating Task (BART)^24^, which is essential for developing trait hierarchies in personality descriptions, as well as for the creation and reinforcement of stereotypes^25^.

Although breadth may appear related to concepts like concreteness or abstractness, it represents a distinct semantic dimension. Concreteness ratings^26^ measure how much a word evokes sensory experience, and modality exclusivity norms^27^ assess how strongly an adjective is linked to perceptual modalities (e.g., vision, touch, smell). In contrast, breadth reflects the behavioral scope suggested by an adjective—how many different types of actions, situations, or contexts a descriptor is considered to summarize. For instance, “punctual” mainly relates to timeliness, while “reliable” signifies consistent performance across various situations. As breadth is not a perceptual or sensory variable but a social–behavioral measure based on attributional processes, cross-situational consistency, and linguistic category theory, we believe it can vary independently of concreteness and sensorimotor strength, which our normings and analysis also empirically support.

To measure adjectival breadth in language use qualitatively, an updated adjective norming database is necessary. However, one currently available database used in research^23^ is quite limited, involving only 573 adjectives and outdated. The current article presents an extensive, updated database with subjective ratings for 1,214 English adjectives regarding breadth and desirability dimensions. Although our rating task framed adjectives as descriptors of human characteristics for clarity and participant engagement, the concept of breadth is not confined to person perception. Breadth, as we define it, is a property of the adjectives themselves: it indicates how broadly an adjective can generalize across behaviors, contexts, and targets. Here are several key highlights of the updated database compared to the existing one. First, the new database is much more comprehensive and includes marginalized terms that are infrequently used in academia but common in everyday conversation. Second, we improved the linguistic interpretation of breadth by defining and contextualizing it rather than merely asking participants to compare them to other adjectives. Third, whereas Hampson *et al*.^23^ involved only *50 raters*, we recruited over 1,800 participants. Fourth, Hampson *et al*.^23^ was conducted several decades ago, while this study captures current interpretations of many longstanding and contemporary terms. Our database not only allows a more detailed semantic categorization for research into the social-cognitive effects of language use but also enables new questions about the behavioral and psychological impacts of adjectives on communication. Practically, the database should support studies in text analysis, natural language processing, and semantic representation related to communication.

## Methods

### Participants

This study was approved by and conducted in accordance with the guidelines and regulations of the IRB at the University of California, Los Angeles (IRB#22-001828). A total of 1,869 participants were recruited and completed the study online through Prolific (https://prolific.co), receiving roughly $12 per hour. All participants were from the United States, native English speakers, and had an approval rate of 95% or higher. Each participant read and provided informed consent before starting. Those who failed either of the two attention checks or did not meet the language-screening criteria were excluded, resulting in a final sample of 1,576 participants. Demographic information was collected from all participants, including age (*M* = 38.3 years, range = 18–81), gender (47% male), race/ethnicity, educational level, and U.S. state of residence.

### Stimuli

The stimuli consisted of 1,214 unique adjectives. The adjective list was compiled through a thorough search of existing academic and publicly available sources. We initially included all unique adjectives from two key datasets^23,28^ related to personality and social cognition. We then added terms from online academic repositories^29^. Finally, to enhance contemporary relevance and lexical variety, we included adjectives commonly used in everyday conversation that are underrepresented in traditional academic norms, including words describing marginalized or niche traits. We avoided free-generation methods because they tend to produce a small set of highly frequent descriptors (e.g., “good,” “nice,” “kind”), leading to sparse and skewed coverage that omits many narrow, broad, or culturally specific adjectives. To ensure a balanced and representative sampling of the full semantic spectrum of adjectives, a curated approach was necessary. Despite the practical constraints of building a norming dataset, our goal was to create the most comprehensive and representative adjective set possible.

### Procedure

After providing consent, passing two attention checks, and completing language screening, participants were asked to read the instructions for the study: “*This experiment concerns the degree to which characteristics vary on two dimensions: breadth and desirability. Some characteristics are broad, implying a great amount of information about a person, whereas other characteristics are narrow, saying very little about the person beyond the specific behavior. We would like to present an example to help your understanding. For example, a person who shows up to a class on time can be described as punctual. This is a desirable (engaging in good behaviors), and narrow (only specific to showing up on time, not above or beyond this behavior) characteristic*.”

To ensure that participants had a clear understanding of breadth and desirability, we defined these terms as follows: *“In terms of breadth: Broad traits are those that refer to a wide range of different types of behaviors, whereas narrow traits are those that refer to a much more limited range of types of behaviors. For each word, you are to decide how broad (abstract, general, global) is its range of behavioral referents. A nine-point rating scale is provided, where 1 is defined as ‘extremely specific (fewest relevant behaviors)’ and 9 as ‘extremely broad (most relevant behaviors)’. In terms of desirability: Please rate how desirable people in general think it would be for an individual to possess the characteristic. A nine-point rating scale is provided, from 1 is defined as ‘extremely undesirable’, and 9 as ‘extremely desirable’.”* During the study, each participant was randomly shown 100 words, rating each one individually on familiarity (yes vs. no), breadth (1–9), and desirability (1–9).

### Data analysis

Ratings of breadth and desirability were included only if the participant indicated familiarity with the word. Therefore, approximately 100 individual ratings were collected for each word on a scale from 1 to 9. By aggregating these ratings, we obtain stable current estimates of these dimensions. For example, the words “good” and “normal” have the highest scores on breadth, while “unpunctual” and “untalkative” have the lowest scores on breadth. Table 1 lists sample adjectives according to their breadth and desirability ratings.

**Table 1:** Categorization of traits by desirability and breadth.

	NARROW	BROAD
**DESIRABLE**	Punctual	Good
	Well-spoken	Fun
	Honest	Creative
	Articulate	Successful
	Studious	Able
**UNDESIRABLE**	Fraudulent	Bad
	Abusive	Horrible
	Misogynistic	Evil
	Horny	Nasty
	Hypochondriac	Sick

In addition, we performed a word-level analysis to explore how lexical properties like morphological structure, negation, and part-of-speech ambiguity might influence breadth ratings. First, we checked whether breadth ratings were systematically affected by morphological structure (e.g., “punctual” vs. “unpunctual”). Using the SnowballC stemmer, we calculated, for each adjective, the difference between its raw word length and its stemmed form, creating an index of morphological complexity. Breadth showed only a very small negative correlation with this measure (*r* = −0.07, *p* = 0.023), and a modest correlation with raw word length (*r* = −0.22, *p* < 0.001). Linear regression revealed that morphological complexity accounted for less than 1% of the variance in breadth (*R²* = 0.004). These results suggest that derivational structure has only a minimal effect on breadth judgments.

We also examined the role of lexical negation, given that prefixes such as *un-*, *in-*, *im-*, *ir-*, *dis-*, or *non-* could theoretically limit the scope of an adjective’s meaning. After identifying all adjectives starting with these negating morphemes, we compared their breadth ratings with those of non-negated adjectives. Although negated forms were slightly narrower on average (≈0.12 points), the effect size was very small (*Cohen’s d* ≈ 0.17), and the difference accounted for very little variance in breadth. This pattern indicates that while negation may have a directional effect, it does not significantly alter the overall structure of the breadth norms.

Finally, we considered whether part-of-speech ambiguity might complicate interpretation for a small subset of items that can function as both adjectives and nouns (e.g., “hypochondriac:”). However, in the rating task, all items were clearly shown as adjectives in a descriptive setting, ensuring consistent understanding across participants. In our corpus-based validations, these items mostly appeared in adjectival uses, further mitigating concerns about grammatical ambiguity.

Taken together, these analyses indicate that potential lexical confounds, such as morphological complexity, negation, and part-of-speech variability, have only a minimal effect on breadth ratings. The normative judgments therefore primarily reflect participants’ assessments of the semantic generality of the adjectives themselves, rather than superficial morphological or structural features.

### Data collection process

The data were collected in two waves by different raters, following identical procedures but using different stimuli due to an unexpected technical issue in Qualtrics. In Wave 1, our study was programmed with the platform’s Loop & Merge function to randomly select 100 adjectives from a full list of 1,214 for each participant. However, after data collection, we discovered that only 952 unique adjectives had been sampled. This problem resulted from a sampling malfunction within the platform, not a design decision on our part. We recruited a total of 1,322 participants in Wave 1, ending with a final sample of 1,097 after applying attention checks and language-screening criteria.

To obtain ratings for the remaining 262 adjectives that were not sampled in Wave 1, we conducted a second round of data collection. Since data from two independent samples would be combined to create the final norming set, we aimed to ensure reliability in sampling characteristics and comparability across waves. Therefore, in Wave 2, we included a random subset of approximately one-quarter of the adjectives from Wave 1 (*n* = 238), along with the 262 missing adjectives, totaling 500 adjectives rated in Wave 2. A total of 547 individuals participated in Wave 2, resulting in a final sample of 479. These overlapping items allowed for a direct assessment of cross-wave consistency. Correlations between Wave 1 and Wave 2 ratings were exceptionally high for both breadth (*r* = 0.90, *p* < 0.01) and desirability (*r* = 0.99, *p < *0.01), demonstrating that ratings remained highly stable across independent samples.

## Data Records

All data are available on OSF at the following link: 10.17605/OSF.IO/2XC8E. Two main data files are in the “adjective normings” folder. The first is a long-formatted, trial level dataset that contains each participant’s ratings for each adjective. The second is an aggregated dataset with word-level averages for breadth, desirability, and familiarity. The “external validation” folder includes the data used for empirical comparisons with existing lexical norms (concreteness, modality exclusivity, and semantic diversity). Additionally, the repository features an interactive Shiny app (with accompanying R Markdown source code) that allows users to generate demographic-specific weighted estimates of breadth, desirability, and familiarity based on selected variables such as age, gender, race/ethnicity, education, and waves of data collection.

## Technical Validation

After the initial data cleaning and processing, various checks and validations were conducted to evaluate the data quality as outlined below.

### Reliability validation

To evaluate the reliability of valence and breadth judgments, we calculated effective reliability using the Spearman-Brown formula^30^. Effective reliability serves as a comprehensive measure of the pairwise correlation stability among raters, reflecting the likelihood that a different group of raters would arrive at similar conclusions about the items^31^. This method is especially useful for assessing reliability across a large group of raters judging multiple items, particularly in novel or challenging judgment tasks^32^. Standard pairwise reliability estimates tend to be low in such rating tasks. Therefore, to examine consistency in judgments across raters, we first computed interrater correlation coefficients over 100,000 iterations, confirming that increasing the number of iterations did not affect the coefficient. Next, we used the Spearman-Brown formula to determine effective reliability. Repeating this process ten times, we found an average effective reliability of 0.99 for desirability and 0.93 for breadth, indicating highly stable estimates for both.

### Consistency

We also conducted several analyses to measure the correlation between our database and the existing ones^23,28^ to assess the new ratings reported here. For the words that were available in Hampson *et al*.^23^, the correlation with British category-breadth ratings (*N* = 45) was 0.75, and the correlation with American category-breadth ratings (*N* = 5) was 0.56. It is important to note that the British and American category-breadth ratings correlated at 0.75^23^. For the desirability ratings, the correlation with British desirability ratings (*N* = 45) was 0.96, and the correlation with American desirability ratings (*N* = 5) was 0.46. British desirability ratings and American desirability ratings were correlated at 0.48. Lastly, our desirability ratings correlated with Anderson^28^ likeness rating at 0.94. Although these comparisons are necessarily limited to overlapping items, the present database substantially expands prior work by including many additional words, including contemporary and previously unrated terms, while maintaining strong consistency with earlier measures. Our new measures are generally consistent with prior published measures, despite being collected in a different social context fifty years later.

### External validation: Comparison of other related constructs

**Fig. 1:**
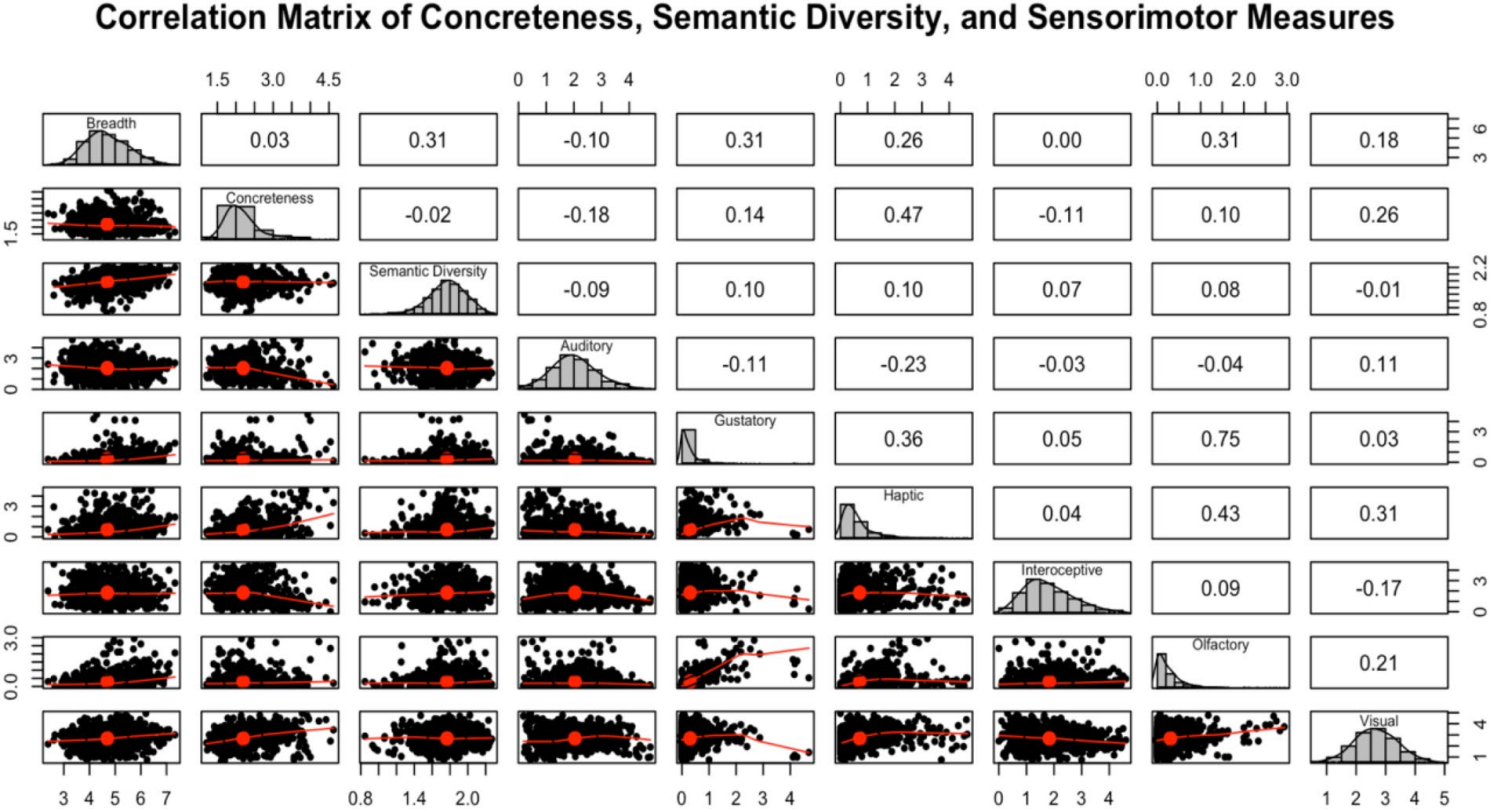
Correlation Matrix of Concreteness, Semantic Diversity and Sensorimotor measures.

To better understand the uniqueness of breadth compared to related psycholinguistic concepts, we compared our breadth ratings with three commonly used sets of lexical norms: (a) concreteness norms from Brysbaert *et al*.^26^, (b) modality exclusivity ratings from Lynott & Connell^27^, and (c) semantic diversity norms from Balota *et al*.^33^ (Fig. 1). We found 1003 overlapping adjectives for the concreteness and semantic diversity datasets, and 929 overlapping adjectives for the modality exclusivity norms. The correlation between breadth and concreteness was very small and not statistically significant (*r* = 0.03, *p* = 0.30), indicating that breadth does not correspond with sensory-conceptual concreteness. Additionally, correlations between breadth and individual sensorimotor modality ratings were modest (e.g., auditory *r* = −0.10; visual *r* = 0.18; haptic *r* = 0.26; gustatory *r* = 0.31), further supporting the distinction between behavioral generality and sensory experience.

In contrast, breadth showed a moderate positive correlation with semantic diversity (*r* = 0.32, *p < *0.01). This pattern aligns with expectations: semantic diversity reflects the variability of contexts in which a word appears, and adjectives with broad behavioral scope (e.g., “reliable,” “good”) tend to be used across a wider range of discourse environments. This moderate correlation supports the idea that breadth relates to, but is not solely defined by, contextual dispersion. Overall, these findings illustrate that linguistic breadth can be distinguished empirically from concreteness, abstractness, and sensory modality ratings, while still maintaining a meaningful connection to contextual variability.

### External validation: Two demos

Two separate validations were conducted using different datasets and evaluation domains. The first validation employed a corpus of instructor reviews from a public course-evaluation platform (https://culpa.info/#/). This source was chosen because its structure provides two distinct reviews per instructor: the most helpful review (with the highest number of thumbs-up) and the most controversial review (with the highest number of thumbs-down). The final dataset included 1,967 professors and 3,934 reviews. For each review, we extracted adjectives using *spacyr*^34^ and assigned breadth and desirability scores from our database. Reviews contained a median of 134 words (range: 1–171) and an average of 5.74 adjectives (range: 1–46). Mean desirability scores ranged from 1.31 to 8.66 (*M* = 6.15), and mean breadth scores ranged from 2.45 to 7.33 (*M* = 5.70).

To assess the reliability of part-of-speech tagging, we repeatedly resampled 1,000 reviews and checked how often words on our adjective list were used as adjectives according to *spacyr*. Across different samples, about 89% of these words appeared as adjectives in the corpus. Next, we built a linear regression model to predict a review’s mean breadth score based on its mean desirability score, the instructor rating, whether the review received more upvotes or downvotes, and all interaction terms. Significant main effects were found for desirability (*β* = 0.21, *SE* = 0.03, *t* = 7.01, *p* < 0.001) and instructor rating (*β* = 0.18, *SE* = 0.06, *t* = 2.97, *p* = 0.003), showing that reviews describing instructors more positively or reviewing higher-rated instructors tended to use broader adjectives. Several interaction effects were also significant, indicating that the link between desirability and breadth depended on both the instructor rating and the review’s endorsement pattern. These results demonstrate that norms of breadth and desirability, based on human ratings, relate to consistent variations in the adjectives used to evaluate people. Overall, these findings reveal that such norms influence the words chosen when describing and judging others in real-world settings.

A brief preliminary report of the second validation has been presented in a conference proceeding^35^. Yet, the full dataset, detailed methods, reliability analyses, semantic comparisons, and external validations appear only in the present manuscript. Here, we briefly summarize the key analyses using a randomly sampled set of 100,000 product reviews from the publicly available Amazon Digital Music dataset^36^. This dataset includes written comments along with 1–5 star ratings from May 1996 to October 2018. For each review, we identified all adjectives and assigned breadth and desirability scores from our database, then calculated average scores for each review. Across reviews, the number of adjectives ranged from 1 to 126 (*M* = 2.24). Mean desirability scores ranged from 1.30 to 8.66 (*M* = 6.95, *SD* = 1.68), and mean breadth scores ranged from 2.61 to 7.33 (*M* = 6.09, *SD* = 0.85).

We fit a linear regression predicting average breadth based on (a) mean desirability, (b) star rating, and their interaction. Both predictors were statistically significant: reviews with more desirable adjectives tended to include broader adjectives (*β* = 0.15, *SE* = 0.008, *t* = 17.18, *p* < 0.001), and higher star ratings were also associated with broader adjective use (*β* = 0.32, *SE* = 0.009, *t* = 35.94, *p* < 0.001). The interaction was also significant *(β* = 0.017, *SE* = 0.002, *t* = 9.14, *p* < 0.001), indicating that the positive relationship between desirability and breadth was strongest in the highest-rated reviews. This moderation pattern aligns with prior observations^37^ that extremely positive reviews often rely on short, formulaic adjectives (e.g., “amazing,” “perfect”), which may be highly desirable but not necessarily broader in behavioral scope, thereby reducing the association at the extreme upper end.

We emphasize that these two validations are not meant to be comprehensive tests of the construct, nor do they cover all the domains where breadth might be applied. Instead, they demonstrate how the database can be used to measure adjective patterns in large, naturalistic text corpora. Since evaluations of people and products differ in their linguistic structure, the agreement observed across these two different domains indicates that our breadth ratings capture semantic information that extends beyond the specific rating task.

Given the wide range of linguistic contexts in which adjectives appear, these validations should be seen as illustrative examples rather than definitive standards. Future research could examine additional corpora, including hedonic versus utilitarian products, opinionated discourse, or areas where negation and hedging are more significant.

Overall, the analysis revealed important variations in adjectives that are fundamental and frequently used in communication. In both datasets, the results show that norms of breadth and desirability based on human judgments align with consistent patterns of adjective use across multiple large-scale, naturally occurring evaluative texts, providing a clear example of how the database can support quantitative linguistic analysis.

## Data Availability

The complete dataset containing norming ratings of breadth and desirability for 1,214 English adjectives is available on the OSF repository^38^.

## Code availability

All codes (analysis and technical validation) were executed in RStudio (version 2023.03.0 + 386). All analysis code used to process and analyze the data is available on OSF^38^. The repository includes (a) the complete R scripts used for data cleaning, aggregation, and effective reliability estimation; (b) the code for reproducing the external validation analyses; and (c) the R Markdown source code for the interactive Shiny application, which allows users to obtain demographic-specific weighted estimates of breadth, desirability, and familiarity. All scripts are annotated to facilitate reproducibility and enable users to adapt the workflows for their own research purposes.
